# Impact of the COVID-19 pandemic on access to the cerliponase alfa managed access agreement in England for CLN2 treatment

**DOI:** 10.1186/s13023-021-02147-y

**Published:** 2022-01-19

**Authors:** Amanda Mortensen, Eva M. Raebel, Samantha Wiseman

**Affiliations:** 1 Batten Disease Family Association, Hamilton House, Mabledon Place, London, WC1H9BB UK; 2 Rare Disease Research Partners, MPS House, Amersham, HP7 9LP UK

**Keywords:** Neuronal ceroid lipofuscinosis type 2 (CLN2), COVID-19, Cerliponase alfa, Managed access agreement

## Abstract

**Background:**

Cerliponase alfa, an enzyme replacement therapy for neuronal ceroid lipofuscinosis type 2 (CLN2), is currently available in England through a managed access agreement (MAA). It is administered every 2 weeks via an intracerebroventricular device. Here we report qualitative research with families of children with CLN2 disease and healthcare professionals (HCPs) who run the MAA, to understand how access to cerliponase alfa via the MAA at Great Ormond Street Hospital (GOSH) in London, and the overall management of CLN2 disease, was affected during the coronavirus disease 2019 (COVID-19) pandemic.

**Methods:**

Telephone interviews were conducted with nine families, representing 11 children with CLN2 disease, and two HCPs in November and December 2020.

**Results:**

Children had received cerliponase alfa treatment for a mean (SD) of 23.1 ± 24.7 months (7.1 ± 4.6 months in the MAA). Families travelled 7–398 km for treatment (mean 210 ± 111 km). Treatment with cerliponase alfa was designated “essential” by GOSH and continued as normal during the pandemic but with extra safety precautions, and no children missed any treatments. Families were highly motivated to continue treatment, despite considerable anxiety about the risk of coronavirus infection from travelling and staying overnight but were reassured by communications from GOSH and the safety precautions put in place. Support therapy services were widely compromised, causing families concern about deterioration in their children’s condition. Families were confused about COVID-19 testing and shielding, and were unclear whether children with CLN2 disease were vulnerable to COVID-19.

**Conclusions:**

Looking forward, advice for children with CLN2 disease should be specific and tailored, taking into account the family unit. Support therapies should be considered essential alongside cerliponase alfa treatment.

## Introduction

Neuronal ceroid lipofuscinosis type 2 (CLN2) is one of a group of rare inherited neurodegenerative and life-limiting disorders, commonly referred to as Batten disease. Mutations in the *CLN2* gene on chromosome 11 cause deficiency in the lysosomal processing enzyme tripeptidyl peptidase 1 (TPP1) [1], which leads to the toxic accumulation of ceroid lipofuscin in neuronal cells in the brain and retina [2]. Children with late-infantile CLN2 disease develop normally in early life but signs such as seizures and coordination difficulties emerge by 2–4 years of age [1, 2]. Disease progression is generally rapid and predictable and by 4–5 years of age affected children have muscle spasms, loss of vision and intellectual disabilities, including difficulty sleeping and becoming distressed for no obvious reason [2, 3]. Most patients are unable to sit unsupported and become non-communicative by 6 years of age, have progressive difficulties with swallowing, constipation, hydration, respiratory function and sleep disturbance, and may need gastrostomy feeding [2]. Children rarely survive beyond 8–12 years [2, 3]. Late-infantile CLN2 is the most common type but around 8% of children present with atypical juvenile CLN2 with symptoms starting around 5–10 years of age [1].

The estimated incidence of CLN2 disease in the UK is 0.78 per 100,000 live births [4]; five or six children are diagnosed with CLN2 disease annually in the UK, with an estimated prevalence of 30–50 children [3].

The clinical management of CLN2 disease focuses on the control of symptoms and complications, using medicines and physical therapy, with the aim of maintaining function and quality of life [2]. Cerliponase alfa, a recombinant form of TPP1—an enzyme replacement therapy (ERT)—is the first disease-modifying treatment for CLN2 disease. It was licensed for the treatment of CLN2 disease by the European Medicines Agency in 2017 [5, 6]. Cerliponase alfa is infused via an intracerebroventricular (ICV) device, which is surgically implanted under the scalp using magnetic resonance imaging (MRI) guidance, 10–14 days before the first infusion [7]. A study in 24 children aged 3–16 years with CLN2 disease reported that treatment with cerliponase alfa significantly slowed decline in motor and language function compared with historic controls over 121 weeks’ treatment [8]. Clinical outcomes with cerliponase alfa have been reviewed by Specchio and colleagues [7].

Cerliponase alfa has been available in the UK since November 2019 under a managed access agreement (MAA) following appraisal by the National Institute for Health and Care Excellence (NICE). NICE acknowledged the benefits of cerliponase alpha seen in the early trials but considered that the benefits of long-term treatment were uncertain [2]. The 5-year MAA was therefore established to collect real-world evidence (until November 2024) for subsequent evaluation by NICE.

Cerliponase alfa can only be administered in a healthcare setting by a trained healthcare professional (HCP) knowledgeable in ICV infusion [2]; at the time of this study, this was only available at Great Ormond Street Hospital (GOSH) in London. Children require the ICV infusion every 2 weeks, which takes approximately 4.5 h [9]. In addition, the MAA requires that children undergo regular clinical assessments at GOSH and that parents/carers complete patient-reported outcome (PRO) questionnaires by telephone. Table 1 lists the eligibility criteria for the MAA, and criteria for staying in the MAA or stopping treatment.

**Table 1:** Criteria for entering and staying in the managed access agreement (MAA) [9]

Eligibility criteria	Confirmed diagnosis of CLN2 disease CLN2 Rating Scale ML score ≥ 2 [10] No other serious life-limiting conditions Patient/carer willing to sign MAA patient agreement Willing to undergo implantation of intracerebroventricular access device
Requirements to stay on treatment	Attend infusion appointments every 2 weeks Attend clinic assessment every 6 months Complete PRO questionnaires every 6 months^a^ Assessments show benefit with cerliponase alfa^b^
Reasons for stopping treatment	> 2 infusions missed in any 14-month period^c^ < 2 hospital assessments^d^ or PRO questionnaires completed in any 14-month period Child is not benefiting from treatment Caregiver wishes treatment to stop Medical reasons

^a^Patient-reported outcome (PRO) questionnaires: Pediatric Quality of Life Questionnaire (Peds QL™), CLN2 Quality Of Life Assessment (CLN2Qol), EuroQol 5-dimension, 5-level questionnaire (EQ-5D-5L)

^b^Evidence of benefit based on hospital assessments and PROs

^c^Excluding medical reasons or public health emergency

^d^Hospital assessments: mobility, cardiac function and speech/language skills assessed every 6 months; eye structure and function, brain structure and function and child development (knowledge, comprehension, social–emotional skill, behaviour) every 12 months; during the restrictions instigated during the COVID-19 pandemic, assessments were deferred or conducted by telephone/video

**Fig. 1:**
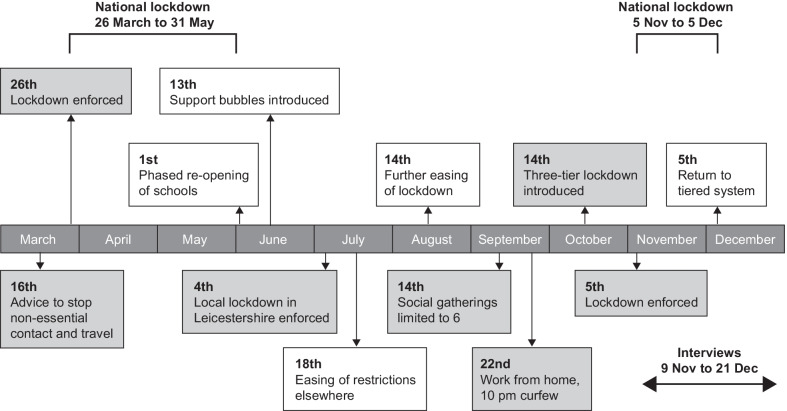
Time course of restrictions implemented during the COVID-19 pandemic in England [11]. ‘Support bubbles’ allowed two households to mix exclusively, without social distancing, under certain circumstances, including families with a child under 5 years of age requiring continuous care and lone parent families [14]. Under the tier system, restrictions in individual cities and regions were determined according to local case rates

The administration of cerliponase alfa at GOSH is potentially burdensome for families, requiring travel to London every 2 weeks. We wanted to understand how the COVID-19 pandemic affected access to the MAA. National lockdowns were implemented in England from 26 March to 31 May 2020, from 5 November to 5 December, and again from 26 December [11] (Fig. 1). Advice on shielding for vulnerable patients considered at particular risk of coronavirus infection was published on 21 May and updated frequently [12]. Rapid turnaround testing for COVID-19 became available in November 2020, aiming to identify non-symptomatic cases [13].

Here, we report qualitative research with families of children with CLN2 disease and HCPs at GOSH to understand how access to cerliponase alfa via the MAA, and the overall management of CLN2 disease, were affected during the pandemic. The insights gathered in this study will help to understand the additional pressures on families during lockdown and will inform discussions with the National Health Service (NHS) and Government agencies about future provision during recurrence of the pandemic or similar situations in the future.

## Method

### Recruitment

At the time of the study, 40 patients with CLN2 disease were registered on the Batten Disease Family Association (BDFA) database, and 18 were enrolled in the MAA. BDFA members who had children in the MAA were invited by email and telephone to participate in this study, in October and November 2020. To be eligible, parents/carers had to be aged ≥ 18 years, have a child in the MAA, and be willing and able to provide informed consent to participate. The two HCPs responsible for administration of cerliponase alfa infusions at the GOSH specialist centre were also recruited.

### Study design

In-depth telephone interviews were conducted by members of the advocacy team at BDFA based on a semi-structured discussion guide developed by the BDFA in collaboration with Rare Disease Research Partners (RDRP), a research organisation that specialises in rare diseases. The guide for families asked about issues affecting them during the pandemic, and their access to treatment. The guide for HCPs asked about issues affecting the specialist centre.

The interviews were conducted between 9 November and 21 December 2020. An interpreter could be made available if needed. All interviews were recorded and transcribed verbatim. Responses were analysed applying an inductive thematic content approach and using the computer Qualitative Data Analysis (QDA) software NVivo. Data were aggregated and remained anonymous. No personally identifiable data were collected, and participants could decline to answer any question and could stop the interview at any point. Permission specifically to use quotes from the recordings was sought, and participants could indicate any content that could not be cited.

This research was conducted in accordance with the British Healthcare Business Intelligence Association's Legal & Ethical Guidelines for Market Research [15]. The survey study was non-interventional with subjects recruited via the patient organisation (BDFA). All participants signed a consent form.

## Results

### Participant characteristics

Nine of 18 families enrolled in the MAA at the time of the study took part in the interviews, representing 11 children aged 4.2–18.7 years (mean 8.9; median 8.8 years) (Table 2). This was similar to the age of the 21 children enrolled in the MAA at that time (5–18 years; mean and median 8.0 years). Ten of the children had been receiving cerliponase alfa for up to 76 months, although one had received treatment for only 1 month. One newly enrolled child was yet to receive treatment. Four children had been receiving treatment with cerliponase alfa for more than 3 years (41–76 months) and five had previously taken part in a clinical trial or expanded access programme. The families lived 7–398 km (mean 210 km) from GOSH. Seven families had one child with CLN2 disease and two families had two children with the disease. All families also had one or two children without the disorder. Further details of families are not reported to avoid potential identification.

**Table 2:** Background information at time of interview (n = 11 children)

	Mean ± SD	Range
Time on treatment (months)	23.1 ± 24.7	0.0–76.0
Time on MAA (months)	7.1 ± 4.6	0.0–12.0
Age (months)	106.5 ± 54.6	50.0–224.0
Distance travelled by families (km)^a^	209.5 ± 110.6	6.5–398.0

*MAA* managed access agreement

^a^n = 9 families

Three interviews were with both parents and the rest with one parent. These interviews took 0:33–3:18 h to complete. One required an interpreter.

The two HCPs responsible for delivering cerliponase alfa at GOSH at the time of the study were also interviewed—a paediatric metabolic consultant and a clinical nurse specialist (referred to as the HCPs). Interviews with the HCPs took about 30 min.

### Effects of the COVID-19 pandemic on the MAA

**Fig. 2:**
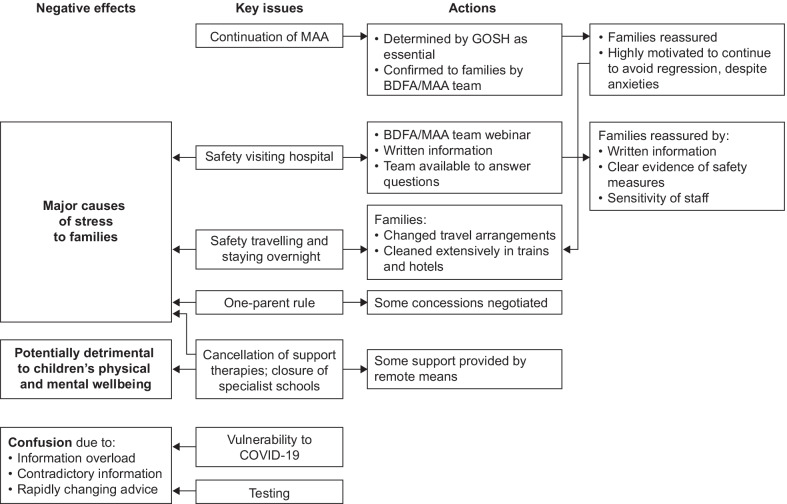
Impact of the COVID-19 pandemic on children with CLN2 disease and their families. BDFA, Batten Disease Family Association; GOSH, Great Ormond Street Hospital (London); MAA, managed access scheme

The key themes describing the impact of the COVID-19 pandemic on the MAA are presented in Table 3, with exemplar quotes from the interviews, and summarised in Fig. 2. The insights included those from one family whose child’s treatment had been delayed because of a cancelled/delayed MRI, and one who said their diagnosis had been delayed because of the cancellation of appointments.

**Table 3:** Families’ comments and concerns about the effect of restrictions on diagnosis and access to the MAA

**Diagnosis**		
1	Delayed diagnosis/tests, and potential effect on child	“A few days later, we were into lockdown, so all of those types of tests and scans were put on hold, so [Child] didn’t actually get his MRI and genetic testing done until… It was July I think, June or July.” “Pre-diagnosis they were going to give [child] an MRI scan. But then, COVID happened and then she wasn’t allowed to have the MRI […]. So we would have known sooner. […] Looking back, you think it’s only a couple of months but a couple of months while [child] regressing so quickly makes a big difference, so potentially we could have learnt that there’s something genetic going on with her because the MRI shows the smaller cerebellum. We could have known that that in June, as opposed to September.”
**Concerns about continued access to treatment**		
2	Fear of losing access to treatment because of hospital closures	“However, I personally was afraid and started imagining thinking that the hospital would be closed down as well. That they would stop the infusions, that we would be asked not to come. But no, luckily nothing happened of that type. And no delay and no cancelation.” “I was absolutely terrified that they were going to stop the Brineura. I’d heard that some of the medical treatments were stopping for people. I was just absolutely terrified that they’d just not offer the treatment at all to our children, knowing how aggressive Batten’s disease is.”
3	Possible delays if child or family required to isolate	“Some concerns […] in terms of if [child 1] had to self-isolate and so on and so forth, whether he would then be delayed access to the therapy. Those were my concerns but we were rest assured straight away that the therapy would continue.” “There should be a process which still allows a child to have [the infusion]. Or is it safe for a child who might have a positive result? Does that have an impact on receiving treatment for them, personally? And so, then you can understand the need to wait, but then, if it doesn’t, is there still a process around being able to access the treatment?”
**Communication**		
4	Families were told by BDFA and by nurses and staff at GOSH that treatment would continue, by email and while visiting GOSH for infusions Families were impressed and reassured by communications about precautions and safety measure put in place, and were not concerned about continuing to attend for infusions	“We got an email saying treatment will continue. I think it was pretty much us asking the question and getting the answer back from Great Ormond Street. Whoever it was there.” “We had a contact from the hospital, presumably from [name] and I had a few emails going backwards and forwards as well. So, yes overall I was very happy with the communication.” “No. I don’t have any. I am really glad or even happy that they keep happening and [CHILD 1] and myself, we just know that whatever happens, that we are going to London every two weeks. That we have to be there. Whatever is happening around us, we just know that we have to go, and luckily we can go, we are going.” “And I thought, well, gosh, we’ll probably have quite strict protocols and guidelines that they stick to and testing and… So the thought of kind of catching it there I felt would probably be quite low.” “I got in touch with Great Ormond Street. They confirmed straight away that things might be a little bit different in the hospital in terms of how many parents could go in, and things like that, but they definitely would not be stopping the treatment because it was a critical treatment. So, there’d be no way that treatment would stop. It might just be that they had to take on extra measures themselves to keep us safe.” “I was told everything that would happen. There wasn’t anything flagged up that I wasn’t already told about. Like I say, the staff have been absolutely amazing and talked us through the whole process and everything. We have managed to build up relationships with the staff so that we knew if anything else had to take place like before and after infusions that we were aware of everything.”
**Hospital visits**		
5	Anxiety due to risk of coronavirus infection	“.., on one of the first visits when we decided to drive because we were panicking about the train, there was an article on the morning that said there’d been an outbreak in GOSH and it was describing how many were infected. And then doctors and nurses were having to isolate and that was a panic because we thought, oh my goodness. It was like headline news and we thought we’re just on our way there now. But actually in fact when we got there we just felt completely safe.” “I think we were quite scared. I was very scared about going to the hospital because we were told that they were using children’s hospitals first to deal with overflow. And so it was a little scary in the beginning how we were going to manage the hospital bed availability and things like that.”
6	Families were reassured by safety measures such as security at door to remind entrants to sanitise hands and put on clean mask; trails marking routes around the hospital, reminders to keep distance	“We felt safe. And then when we got there the nurses were so lovely and so reassuring and extra nice and extra sensitive because they knew we were all in a bit of a panic and like oh God, it’s the first time we’ve come down and we’ve made it.” “It just put my mind at ease straight away, as soon as I spoke to them.” “Every time I’ve gone, there’ve been at least two security guards on the door… And sanitise your hands as well. Once you’re on the ward, it’s a little bit different, and you really can take your mask off but walking through the actual hospital that is… It was just treatment as normal with extra precautions.” “when you walk in, there’s the cleaning station, sanitising station, there’s usually somebody on the door. […] I can see there’s a difference there as you walk in. There’s the routes. You’ve got the left-hand side routes through that they can see your route through the hospital is marked, a little bit.”
7	Families were reassured by witnessing nurses changing PPE and washing hands and the ‘business as usual’ attitude	“Yes. I’ve noticed that the staff, whoever I’ve got contact with, mostly nurses, they do comply to the new procedures, and I can see them wearing and changing and really changing PPE. And washing hands and remembering and keeping distance as much as is possible.” “The nurses are amazing and the atmosphere hasn’t changed. Everyone is lovely and friendly and you don’t get the impression that anyone is worried up there. It’s all business as usual and it’s been just as fun for [child 1] as ever.”
8	While staff were in PPE, they did their best to ease distress to children by wearing patterned masks and coloured scrubs	“You just soon got used to them putting the masks on and they had normal masks. And then I think some of the colleagues had made ones with brighter colours and brighter patterns because some of the children were getting distressed because they couldn’t see the person and it looked a bit foreign and a bit alien. […] later on in the lockdown …they got different scrubs and they had pink on and blue and tried to make it as friendly as they could for the children, so that was nice… She was in the full top to toe pink and she had a Disney lanyard and all this, so it was lovely.”
9	One-parent rule was challenging	“It was hard[er] to get used to the one parent rule. They were kind to us and they did allow us to be together at points, because we had a new diagnosis.” “I was there on my own with both children when they had the brain surgery. […] I’ve always been down in recovery with [child 2] when he comes out of any type of surgery or when he has been put to sleep. I couldn’t do that at Great Ormond Street.” “In terms of safety, from the hospital, I didn’t have any concerns. It was harder to get used to the one parent rule. They were kind to us and they did allow us to be together at points, because we had a new diagnosis” HCP: “If they have more children, then who are they supposed to leave the children with? I think we need some concessions occasionally, to certain families, in certain circumstances. But otherwise, yes, there was the rule, and I think it still is the case, that only one carer can come in.” HCP: “During the pandemic, having one parent there is obviously completely difficult for parents, and other children were off school as well. And they were trying to isolate as much as possible and trying to gain childcare for their other children. We understand that’s completely difficult as well for them all.”
**Travelling and accommodation**		
10	Extreme anxiety using public transport	“Anxiety, particularly, for us with child 1. You want to stay out of the way and reduce your risk, but we still had to get on a train to London every fortnight. I think that was our biggest, and still is, perhaps, our biggest potential exposure. […] and the fact that you’re going through a railway station, two railway stations, sitting on the train.” “We were too frightened to get on the train in the first lockdown. […] I felt like when we first were going down there, I felt like my heart was beating too fast. I don’t know. It’s really hard to explain because I’ve never felt anything like this before. […] I think it’s just because I’d got myself so wound up and anxious. I was thinking, right, if I don’t take her, she could die. If I do take her, she could die.” “Absolute nightmare. At the very beginning, we thought right, we’ll go by train and then when it was in the height of it all and we just thought, oh God, no. And then I started getting really anxious. I started crying on one of the journeys home. I just felt completely on a different level of anxiety and paranoia and I was like who are they and why are they sitting here? And they shouldn’t be travelling. They could’ve been a doctor or they could’ve been anybody but I was just like well why are they sitting next to us? And then I started crying and I couldn’t, it was awful. I just didn’t want the children to breathe or move or do anything on the journey up.” “Probably, the risks are there, aren’t they, in terms of catching Coronavirus but I’ve kind of just accepted that that’s the only way we can really get there.”
11	Concerns about putting other family members at risk	“It made me feel like I was putting my dad at risk, even my mum, being in the house because we were travelling to London, which is quite a hotspot for COVID. I know at one point, GOSH did have COVID patients in it, children that had COVID. […] I just felt like I was putting my dad massively at risk, but the risk of [child 1], something happening to child 1, felt greater. So, I felt a bit stuck between the two, but there’s no way I could have not taken her for the treatment.”
12	Accommodation	“We stayed in serviced apartments […] That was all right but again that was stressful because we went in there and we cleaned everything and we didn’t touch the handles and we would tell the children, don’t touch that. And the communal lifts like, don’t touch the lift buttons and that […] so that was quite difficult.” “[…] we thought staying in patient accommodation was too risky. So, we were getting up at 3 in the morning, driving down there, child 1 was having her infusion.” “…have to feed him in the room because there is nowhere suitable for him to sit. You are going backwards and forwards to the kitchen and if there is someone in there you are not supposed to go in. You are not supposed to go and get your stuff out of the fridge. So, technically speaking, I would have to wait perhaps 20 min before I could even go and get the fruit out of the fridge to take back to the room for him to eat. You can’t really hurry [child 1] with his eating.” “Again, you get into that routine. Even when we get to the hotel, and we stay the night before, the first thing I do is sit child 1 on the edge of the bed, and go around with the wipes. It’s not that I don’t think they do a good job of cleaning the place. They do. It’s just in the back of your mind, you just think, I’m happy if I go round and clean all the surfaces that child 1’s likely to touch, now. I’ve got that, so I can forget it, then. So, it’s things like that, that we do. We manage them [concerns] by our cleaning frenzies when we get there or get on the train” “Yes. Well, we used to get on the train and we kept the children in the buggies. And me and [Name] both had a set of [bleach] wipes and a set of sprays, so we would go and spray the seats, the windows, the curtains, the handles, the doors, every single thing in that little bit of carriage where we were going to be sitting. So that was stressful enough because you’re already anticipating, oh, I can’t just go and sit down.” “It was just pretty plain sailing when we went down on the train. It wasn’t as stressful as I thought seeing the staff had thoroughly cleaned, but I think it was just me and my worries which was the reason why I cleaned it again” “And the best options for me to continue [CHILD 1] infusion going to London, was by car, to get there by car to avoid public transport.” “I was so anxious, we decided we would drive and yes, it was a nightmare. We felt safe because we thought, oh well, we’re going literally from our house, opening the car door, we’re opening the car door again in London and going straight to hospital.” “We used to get a taxi when we arrived down there because we get in at half-past nine in the evening. We used to get a taxi from Kings Cross down to Western, but we don’t do that now. We walk.”

Treatment under the MAA is delivered by a small nurse-led team. Admission for surgery to implant, replace or remove the ICV device, and for administration of the infusion, was classified as an essential service following discussions between the HCPs and management at GOSH, and could therefore continue during pandemic restrictions. Additional staff were trained to administer infusions should any of the established staff become unwell or have to isolate, although in reality this was not required. The registrars within the metabolic team were redeployed to other areas, meaning that unfamiliar doctors worked with the MAA team at times.

The interviewed HCPs described the situation as “treatment as normal with extra precautions”. Two children underwent surgery (one to implant the ICV device) and no infusions were cancelled or delayed (including one child whose family were isolating); however, assessments were postponed for some patients. One HCP commented that they had not thought about the impact of the pandemic in any depth until the interview. One was proud that the team and colleagues had been able to continue the MAA, including starting two new patients on treatment.

Families reported being anxious about whether the infusion programme would continue during the pandemic and about missing or delaying an infusion because of the need to isolate (Table 3, rows 2 and 3). Some were concerned about implantation of the ICV device but were nevertheless happy that their child would receive treatment.

Two families reported that MRI scans and genetic tests were delayed by several months early in the first lockdown, delaying the diagnosis of CLN2 disease and causing concern about potential progression of their child’s condition during this time (Table 3, row 1).

Families were told by the BDFA and the MAA team that treatment would continue—by email and during hospital visits for infusions. Families reported that the main reason to continue with treatment was the potential regression of their child’s condition if they were to stop infusions, and they felt that the benefit outweighed the possible risk of infection with COVID-19. Families felt reassured by the nurses at GOSH that the environment was safe, and appreciated the extra sensitivity of the nurses to their concerns. Generally, families were relieved that treatment could continue; one described being able to receive treatment through the MAA as “comforting”.

#### Communication within and from the hospital

The HCPs felt they were kept well informed by GOSH, with daily briefings on COVID-19 and staffing rates, and constant updates on local and national guidelines on, for example, use of personal protective equipment (PPE) and patient access to the hospital. The HCPs also described concerted efforts by GOSH to inform families about hospital policies during lockdown. The MAA clinic team ran a webinar for parents in collaboration with the BDFA. Clinicians and the nursing team also sent regular email updates, and clinicians were available at all times to answer questions. Families reported being impressed and reassured by communications from GOSH about precautions and safety measures during access for the MAA (Table 3, row 4). Issues around communication of broader issues are described in “COVID-19 risk and shielding” section.

#### Safety while accessing the hospital

The HCPs reported measures implemented by GOSH to ensure the safety of staff and patients by reducing contact, such as working from home where possible, use of PPE, hand washing and social distancing. Many clinics were run by telephone or video, but some were cancelled; only a few selected patients were seen face to face. Additional safety measures at GOSH included security at doors and reminders about social distancing and to use a clean face mask on entry; trails were established to help patients navigate around the hospital.

Families described high levels of anxiety about visiting the hospital and the possible risk of coronavirus infection (Table 3, row 5) but also reported being reassured by the safety measures implemented at the hospital (Table 3, row 6).

HCPs were aware of families’ anxiety about attending hospital and the risk of contact with other people and described reassuring families that precautions were in place to protect them. Families were reassured by witnessing nurses changing their PPE and washing hands and the ‘business as usual’ attitude (Table 3, row 6). They appreciated efforts by hospital staff to minimise distress to children caused by PPE, for example by wearing patterned masks and coloured scrubs (Table 3, row 7).

A one-parent rule was implemented, meaning that only one parent could accompany a child to hospital. Families reported finding this rule challenging in terms of organizing childcare for siblings at home, being unable to leave their child with CLN2 disease while attending to other matters (such as organising reimbursement for transport), and the difficulty of managing a child in a wheelchair (Table 3, row 9). HCPs also commented on the challenges that the one-parent rule presented in terms of childcare for siblings, especially when schools were closed. They described making accommodations to the rule where physically and sensibly possible, such as for families attending for their first infusion or surgery.

#### Transport and accommodation

Some families travelled long distances to GOSH to receive treatment under the MAA: five travelled more than 200 km, with the longest journey of 398 km (Table 2). Families described being extremely anxious about using public transport (Table 3, row 10) and staying in overnight accommodation in order to attend GOSH for infusions (Table 3, row 11). Concerns included the risk to other family members (Table 3, row 10), the effect of potential disruptions to public transport, the risk of infection at the hospital, meeting their child’s nutritional needs while away from home, using shared facilities in patient hotels, and accessing shops (Table 3, row 11). GOSH advised families to take extra precautions against infection when travelling, to protect both themselves and the hospital staff. Families described cleaning surfaces on trains and in their rooms, and avoiding touching anything in communal areas. They acknowledged that their anxiety was worsened by travelling but that cleaning made them feel better (Table 3, row 11). One family resorted to doing the long journey from home to GOSH and back by car in one day, leaving at 3 am, rather than staying overnight. Others travelled on trains at quieter times, maintained social distance on public transport, and drove or walked in preference to using taxis.

#### Assessments

Whilst infusions could continue as normal, MAA-related assessments (e.g., brain scans, psychology assessments, speech and language assessments, physical assessments, eye assessments) were largely cancelled or delayed; however, it was agreed with NICE that face-to-face CLN2 scoring would continue to assess whether ERT was effective.

### Impact on the care of children with CLN2 disease

Families reported broad-ranging impacts of the pandemic on the care of their children during the pandemic (Table 4), particularly the cancellation or disruption of therapy services (e.g., massage, physical activities, speech and language therapy, physiotherapy) (Table 4, row 1) and specialist schools (row 2). Several families perceived a deterioration in their child’s condition and described the difficulties of supporting their child without these services or advice from them (Table 4, row 3). Families also voiced concerns about being separated from their child/ren should they be hospitalised, and whether their child would receive adequate tailored care in hospital without a family member on hand.

**Table 4:** Effect of the pandemic on overall care

1	Cancellation of support services	“Our primary concerns immediately were the fact that all his therapies that he relied on and made his week what it was where just stopped overnight. So, immediately that was our concern.” “We were just actually starting with the chiropractor. […] And we had an appointment and then that was cancelled and we were going to do that regularly with her to go to a massage clinic, well it’s called a Wellbeing Centre, so we were looking forward to doing that. But that hadn’t started so that was something that she wasn’t missing, she just hadn’t started.” “The physiotherapist from [CHILD 1] school, that there is really no contact whatsoever with them. And I really feel that [CHILD 1] is missing a lot as a result of not having, me not having contact, me doing nothing with him. Not knowing what to do with him at home.” “One very specific thing is going to back to the physiotherapist at [CHILD 1] school. […] I don’t have high expectations of what they do daily something with [CHILD 1], but at least to give me something, some exercises, examples that I could do with [CHILD 1]?” “The main lockdown of [swimming facilities] being shut and [School] being shut, she was just here with us, wasn’t she? And can’t access like anybody else would’ve. You can’t let her go online on her own, things like that, whereas, other children, other young adults, would.” “Then what’s not worked is just the lack of professional involvement from, well, it’s been across the board. Not having that input that we need more so with them regressing more. Me not being able to have a break. I’m going into robot mode at the minute.” “The 7 months of therapy that everybody has lost because obviously you can do things like Zoom and different bits and pieces but the hands-on therapy… it’s a lot of story massage, […] so it’s a therapy class so it’s music therapy. It’s massage therapy, it’s Rebound Therapy, it’s hydro, it’s physio, so that’s five, six hours all day she’s getting good stimulation in what she needs. Obviously, at home we do the same but you can’t do as much as they would do at school. […] It was the physical things and having the physios on-site to look at the children every day.” “It was making sure that the physios and OTs could keep up what they were doing and keep up with their progress just so they could support them […]. Things in the house like the stairlift to become unsafe. I needed to make sure that I could keep them safe around the house so I didn’t have a choice but to get people involved and have people round. […] Even though it was scary, things like coming to the house and they wore full PPE. I did make sure that I wiped down with anti-bacterial wipes after so that adds more pressure on me after having more people around.”
2	Closure of specialist schools/respite care	“[Child 1] has got a gastrostomy and she needed to get it changed, so we had to have the nurses come out to the house to change it. So they came in at the door, they put full PPE on at the door, you know visors, gloves, aprons, and then [Name] is actually trained in it thankfully so they had to just come to observe. […] and signed him off. And so that was new because that would just have got done at the school. But thankfully that could be done at home so it was all very accommodating.” “One very specific thing is going to back to the physiotherapist at [CHILD 1] school. […] I don’t have high expectations of what they do daily something with [CHILD 1], but at least to give me something, some exercises, examples that I could do with [CHILD 1]?” “I think it’s the childcare aspect. I think, for me, just the hardest thing of this season is that I’ve been [name] main carer. […] has made it harder to call on people for help because they’re not allowed in your home. And so, for me, personally, I’ve felt quite exhausted because there’s been no stop, no breaks. It’s full-on, and I think that’s probably the hardest thing that’s been drawn out of this.” “The main lockdown of [swimming facilities] being shut and [School] being shut, she was just here with us, wasn’t she? And can’t access like anybody else would’ve. You can’t let her go online on her own, things like that, whereas, other children, other young adults, would.” “Then what’s not worked is just the lack of professional involvement from, well, it’s been across the board. Not having that input that we need more so with them regressing more. Me not being able to have a break. I’m going into robot mode at the minute.” One family received 2 h’ every other week, where someone came in the house and watched the two children with CLN2 disease so the parents could have time by themselves or spend one-to-one time with the third child and bring the child somewhere. During COVID-19 this help stopped. “Even though it wasn’t a lot, it was a designated time where we could just think, right, we know for a fact we’re going to get a break.”
3	Detrimental effects on children	“I just think when we were all completely locked down, […] child 1 seemed to deteriorate such a lot with not having any physio, not having much to stimulate her mind and things.” “From all of his therapies, he declined. We saw him physically decline in speech, core stability, being able to go from sitting to standing. All these things just stopped. He stopped walking. He was having someone to hold on to. All this happened during lockdown. I do believe though if his horse-riding had carried on and his swimming, and his conductive education, and his speech and language and music therapy, if he had all of those things and he carried on going to school he would be in a much better shape now than back then. Actually, we have seen a real upturn within one or two sessions of him starting conductive education in September. He took about eight steps walking unaided. It’s having that professional expert input which is so important.” “I definitely think, during that big lockdown, that she lost a lot of skills, communication skills, motor skills, mobility. I just felt like I was watching her just deteriorate in front of my eyes. […] I bought all sorts. […]I just felt like I was watching her go downhill. I do feel better now that she can go to school.”
4	Virtual meetings were unsatisfactory for some whereas some saw benefits	“We had the Zoom kind of diagnosis meeting on the Wednesday, […]another Zoom Meeting on the Thursday “Even now, we still haven’t ever met her paediatrician face-to-face. That was one of the impacts of COVID that we were under investigation, but never actually got to see the paediatrician” “[Child’]s eye ophthalmology test, it was done over the phone which wasn’t ideal and obviously it’s not in-depth at all.” “Everything else was done in Zoom calls or we just emailed. So it strips away the unnecessary things that you normally do […] finding a car parking space, stressing to get the wheelchair out […] It’s just far more effective and efficient.” “And the most helpful or grateful thing is definitely the meetings on Thursday, the Zoom, the opportunity to get to know other families who are experiencing or go through similar experiences.”
5	Cancellation of assessments	“He was supposed to do a sleep study but we’ve delayed it now even further because of lockdown […] There was a urology appointment that he was supposed to have which, obviously, that got stopped as well […]it wasn’t a huge problem or affecting his health. There wasn’t anything where we felt he really needs to have this now.”

Some families reported anxiety about obtaining medicines, delays to prescriptions for standard medicines, being challenged about the frequency of prescriptions, and unexpected changes to formulations of some medicines.

One family with a newly diagnosed child were unable to meet their paediatrician face to face. Others reported delays in specialist appointments (cardiac studies, sleep studies, urology) and one commented on the inadequacy of an ophthalmology assessment done by telephone (Table 4, row 5). While some families found contact largely by video frustrating, others felt that it saved a lot of the stress associated with hospital visits (see Table 4, row 4). One family particularly appreciated weekly video calls organised by the BDFA for families to share their experiences.

### Broader COVID-19 issues affecting families

#### COVID-19 risk and shielding

Information was provided/available to families from Government (e.g., via websites), local authorities, GOSH, leaflets, the media, internet, schools and video calls with other parents of children with CLN2 disease. The only specific advice families reported receiving was that children in general were less affected by COVID-19 than adults, and that vulnerable children were not at increased risk of infection. Families were also advised to stay local. Only two families reported receiving information from Government, and two sought information from the media. One respondent said they would have liked to receive personalised information from any of the services involved in the regular care of their child (besides GOSH). Families valued the parent guide to the MAA published by RDRP [9] and the availability of the BDFA’s telephone helpline. They also found social media groups useful during this time.

Families reported concerns about the risk of coronavirus infection to their child/ren and the rate at which it was spreading. They described feeling overloaded by the amount of information in the media, which was often contradictory, and families were unclear about how vulnerable their children with CLN2 disease were to the infection. One family felt that their child/ren were vulnerable because of respiratory problems and were concerned that they would not tolerate ventilation if they contracted the virus. More than half the families said they did not receive specific advice about shielding and therefore made their own decisions. The HCPs also reported that families had been confused and anxious about the Government's advice to shield that was issued after they had received a tailored letter from GOSH. The HCPs advised families who contacted them that shielding was not necessary for their children with CLN2 disease, although providing specific advice became more complicated during the tiered lockdown (see Fig. 1). Two families valued reassurance from the child’s clinician or family doctor that their child was not at increased risk, and one family said they were not any more worried about coronavirus than about infections in general.

To manage their anxiety, families reported paying less attention to the media, and adjusting to a ‘new normal’. Whilst five families adhered strictly to government guidelines, others reported weighing up the risks to the family as a whole against the benefits to their children of attending school and social contact. Some families reported feeling less frightened during the second lockdown than during the first.

#### COVID-19 testing

Eight children with CLN2 disease or a family member had been tested for COVID-19 at the time of the interviews but with no positive results (one family was waiting for results). Families voiced concerns about the discomfort of the test procedure, its accuracy, and the value of the result (e.g., they could become infected soon after having a test). However, one family were not concerned “given the grand scheme of things”. Families reported concern about the lack of information regarding what to do in the event of a positive test. Families were also concerned about the advice that testing was not necessary if families were isolating.

### Effects on families and carers

Families described additional stress due to loss of family support systems because of travel restrictions, social distancing requirements and vulnerability of family members. A lone parent noted feeling particularly isolated. However, another family felt that little had changed as they received little support in the first place. Families described the pandemic as adding extra difficulty to a complex situation. They described feeling abandoned, isolated and alone, lacking time for self-care, and noted the return of feelings of injustice. Siblings were also affected, especially those who were required to isolate because of their vulnerable sibling, adding to their stress. Parents reported being exhausted by being their child’s sole carer: “I think it’s the childcare aspect… for me, just the hardest thing of this season is that I’ve been [name]’s main carer. [Lockdown] has made it harder to call on people for help because they’re not allowed in your home. And so, for me, personally, I’ve felt quite exhausted because there’s been no stop, no breaks. It’s full on, and I think that’s probably the hardest thing that’s been drawn out of this.”

Families felt that there was little respite in caring for their children with CLN2 disease during this time, as services were compromised by COVID-19 restrictions. One family commented on the loss of respite services, which they received for 2 h a fortnight, and two families did not get expected referrals to the hospice. Families also commented on the difficulties of managing their disabled children without support services and the disrupted access to specialist schools.

The HCPs were fully aware of the difficulty of the overall situation for families, and the emotional impact, on top of caring for a child with a severe disability, particularly on those with a new diagnosis and those starting treatment. They noted families’ anxiety around travelling, inconsistent advice about shielding/vulnerability, and attending hospital in general—which they felt applied to many families attending GOSH.

## Discussion

A global survey by EURORDIS-Rare Diseases Europe of more than 5000 patients and their families representing 933 rare diseases in April 2020 found that 90% had experienced interruptions to care; almost 30% of patients who usually received care in hospitals reported that their usual hospital or unit was closed [16]. Our qualitative survey with the families of children with CLN2 disease describes marked impacts of the pandemic on access to support services and family wellbeing. This coincides with recent studies reporting on the extensive worldwide disruption that paediatric and adult patients with inherited metabolic diseases have experienced during the COVID-19 pandemic [17–20]. Our key recommendations to address these impacts in the future are presented in Box 1.

**Box 1:** Key recommendations

Identify specific vulnerability by cross-functional experts in the disease and communicate to families as soon as possible Quickly clarify any contradictory general Government advice Tailor advice to family units, not just patients Designate support services as essential Re-evaluate the advantages and disadvantages of the one-parent rule	

Administration of cerliponase alfa via the MAA, including surgery to implant the ICV device, was designated an essential service and therefore continued, despite widespread reductions in other services in the NHS and redeployment of staff to care for patients with COVID-19. The two HCPs reported running the service "as normal; but with extra safety precautions in place" –a commendable achievement given reports of widespread disruptions to the care of children with rare diseases, including administration of ERT [16]. The continuation of ERT with cerliponase alfa is in contrast to ERT for other lysosomal storage diseases: surveys among patients in Turkey [21], Italy [22] and Spain [23] reported disruption to hospital-based ERT, although patient anxiety about attending hospital for treatment was a major contributing factor. Reassurance from GOSH and the HCPs that families could safely visit hospital may have contributed to persistence with cerliponase alfa during the pandemic, alongside families’ determination to continue treatment despite the difficulties.

The registrars within the metabolic team were redeployed during the pandemic, meaning that unfamiliar doctors worked with the team. Given the rareness of these metabolic diseases, and the essential nature of the treatment, it is reasonable to recommend that some specialist registrars remain with the metabolic team to ensure continuity of care for children with CLN2 disease.

Patients involved in the NICE appraisal of cerliponase alfa described the emotional, physical and financial impact of CLN2 disease, especially when parents often become full-time carers [2]. These parents felt that cerliponase alfa treatment had halted the deterioration in the child/ren’s health, with immeasurable benefits on their family lives [2]. They also considered the burden of travelling to be insignificant compared with the benefits of treatment to their children [2]. The families interviewed in the current study were clearly determined to continue cerliponase alfa treatment during restrictions, despite extreme anxiety about travelling and attending hospital, as they considered that the benefits of the treatment outweighed the potential risk. They made great efforts to modify travel arrangements and felt reassured by information from HCPs and evidence of safety measures put in place by the hospital. Nevertheless, some families travelled long distances to receive treatment (up to 398 km; mean 267 km [Table 2]), as GOSH was the only specialised centre at the time. Administration at Manchester Children’s Hospital is now being set up to ease travel pressure on families.

The original conditions of remaining in the MAA included regular hospital assessments (Table 1), whereas these assessments, other than CLN2 scoring, were not conducted unless they could be done remotely. However, children remained in the MAA even though the criterion for attending hospital assessments was not met, as provision was made in the conditions for national health emergencies. CLN2 scoring and PRO measurements continued and together were used to assess the continued benefit criterion; however, insight into some of the specific effects of cerliponase alfa may have been lost.

Whilst cerliponase alfa treatment continued, children with CLN2 disease also require a wide range of support services such as physical therapies, chiropractic, massage, and speech and language therapy; however, such services were largely stopped during the pandemic. These services are often provided through specialist schools, but these were also closed. Families reported declines in their child’s function/ability without this additional support (Table 4, row 3), although they did see improvements once services resumed. Some parents received video support but felt unable to give their child as much support as a specialist school. Thus, input from professional experts was considered vital. Whether the lack of physical and support therapies has had a detrimental effect cannot yet be known. It is important that overall care requirements are considered when determining which services are essential, and that therapies that are key to patients’ physical and mental wellbeing are maintained in addition to access to medical treatment.

These effects of the pandemic on services are consistent with a recent report by the Genetic Alliance UK, based on the EURORDIS Rare Barometer Covid-19 Experience Survey and weekly community meetings during April–June 2020 [24]. That survey identified widespread disruption to services and support for patients with rare diseases: closure of units, loss of equipment seconded to care for patients with COVID-19, disrupted access to medicines, and loss of support from neighbours, family, psychological services, home care, respite care and day care. Two-thirds of the respondents reported that these changes had probably or definitely been detrimental to their wellbeing, more than half reported detrimental effects on their health, and one-fifth that changes were potentially life-threatening [24]. The Action for Rare Disease Empowerment has recently called for treatments and therapies for rare diseases that are essential for maintaining minimum health to be recognised in formal guidelines, especially if withholding such treatments may lead to irreversible impacts on health and quality of life; such treatments should not be considered ‘elective’ [25]. Thus, support services for children with CLN2 disease should be considered ‘essential’ for those who rely on them to maintain their physical health, to ensure that patients do not deteriorate. Adequate capacity and resilience within such services is vital to ensure that care can continue with staff absences. Support for patients and carers is also vital to help maintain patients’ and carers’ physical and mental health.

A key challenge for the interviewed families was understanding the vulnerability of their children with CLN2 disease. Conflicting information in the media was compounded by blanket government advice about shielding. Patients considered ‘clinically extremely vulnerable’ were advised to shield and were eligible for Government and NHS support, whereas those considered ‘clinically vulnerable’ were advised to stay at home as much as possible but were dependent on family and volunteers for help [24]. However, letters from Government confirming the ‘clinically extremely vulnerable’ status came late, or not at all; different sectors were covered unevenly, and people with the same condition received different advice in different parts of the country [24]. Furthermore, the reasoning behind the categorisation of conditions and risk was not clear, and decisions were changed frequently and often suddenly [24]. In line with the report by the Genetic Alliance UK, the interviewed families described confusion about whether to shield or isolate, particularly when they had school-age children or continued to work.

Patients with children with rare disease are often managed by multiple specialists and are therefore potentially at risk of receiving contradictory advice [26], as seen in our study. HCPs in the MAA reassured any families who contacted them that children with CLN2 disease were not at increased risk from COVID-19. It is therefore important that, in a future scenario like the pandemic, the risk for individuals with particular conditions is determined by appropriate experts, and that the same advice is communicated clearly and consistently to all patients with that condition. It is also important that advice considers family units rather than individual patients, addressing issues around siblings, schooling, working and caring for affected children, and the impact of shielding/isolation on the physical and mental wellbeing of families. Whilst it may not be possible to advise for all scenarios, patients and families should at least feel equipped to make informed decisions.

Families were also confused about advice around COVID-19 testing—when this was necessary, who should be tested, and how to access tests. This was a widespread issue early in the pandemic but is now resolved since widespread testing became available late in 2020. However, the situation again speaks to the need to provide coherent advice that is tailored to families with specific health issues rather than blanket advice that many people find difficult to interpret.

The one-parent rule for hospital visits caused issues for some parents in terms of childcare for siblings and when visiting the hospital. The HCPs also commented on difficulties with the rule that they witnessed but reported making some exceptions after discussion with senior hospital decision-makers, particularly for new patients on the MAA. However, this particular rule seemed to cause a lot of anxiety for parents. Whilst the rationale is understandable in terms of minimising contact, decisions such as this need to be considered in a broader context, and reviewed regularly, including weighing the potential stress and anxiety for families and children receiving treatment against the risk of infection. Use of PPE and social distancing could mitigate the risk.

A potential limitation of this study includes the use of qualitative data, which may be biased by the interviewer during data gathering or during the interpretation of the results. These issues were minimised by using a semi-structured interview guide during interviews, which were always undertaken by the same three BDFA employees. Furthermore, data were analysed using a structured computer software by a researcher who had not been involved with the interviews hence avoiding pre-conceived themes within the responses. Qualitative research is indeed useful to obtain real-world insight from the perspective of the subject rather than the researcher, particularly as open-ended questioning allows issues to be explored in detail and new ones identified [27], hence a strength from this study lies in the in-depth nature of the data reported by the families. The study was also limited by its small sample size, resulting from the rarity of CLN2 disease and the families that were enrolled in the MAA at the time. Nevertheless, although this study was small, 50% of patients participating in the MAA at the time of the study undertook the interviews, providing broad important insights into the overall management of children with CLN2 disease during the pandemic, such as the detrimental effects of closures and disruption to support services and special schools, which clinicians may not be aware of. It also identified effects on families accessing the MAA during the pandemic such as challenges and anxiety associated with travelling to GOSH and staying overnight. This insight into the effects on the family unit, rather than just the patient, is important when considering complex and severely debilitating conditions in children. A further strength of this study is the additional perspective provided by the HCPs involved in the MAA, providing insight into the challenges of continuing the MAA in a safe way and working with families to ease anxiety.

## Conclusions

Administration of cerliponase alfa via the MAA was able to continue as an essential service throughout the pandemic during 2020, thanks to the advocacy and commitment of the HCPs at GOSH. While hospital visits for infusion were stressful, families were strongly motivated to continue treatment and were reassured by the safety procedures and communication about these from the HCPs at GOSH. However, loss of support services and lack of coherent advice about shielding and testing added to families’ anxiety. We therefore make the following recommendations for the future.

- The risk and vulnerability of individual cohorts of patients (i.e., with a particular condition) should be determined as soon as possible, and by HCPs with specialist knowledge and face-to-face experience of affected patients and their families. All specialists involved in the care of the patient cohort should be consulted, to avoid contradictory advice.
- Advice should be tailored and consistent, take into account family units, and provide adequate information to empower families to make informed decisions. Any potential confusion arising from blanket government advice should be clarified by the specialist HCPs.
- Expert support services should be designated essential for patients whose mental and physical wellbeing is highly dependent on them and should continue running with adequate safety measures in place. If services cannot continue, parents require support and guidance to help children at home, in order to mitigate potential deterioration in their condition.
- The one-parent rule for hospital visits should be reappraised, balancing the emotional and practical difficulties for families against the risk of infection.

## Data Availability

The datasets generated and/or analysed during the current study are not publicly available due to patient confidentiality.
